# Exchange interaction for the triplet superconductor UTe_2_

**DOI:** 10.1038/s41598-023-41376-y

**Published:** 2023-08-26

**Authors:** Chih-Kai Yang, Chi-Hsuan Lee

**Affiliations:** Graduate Institute of Applied Physics, National Chengchi University, Taipei, Taiwan, Republic of China

**Keywords:** Materials science, Physics

## Abstract

UTe_2_ is one triplet superconductor that defies conventional relation between ferromagnetism and superconductivity. Our search for a theoretical explanation starts with one spin-triplet state of two electrons and construct a two-particle exchange interaction that favors the formation of Cooper pairs under the configuration. A modified application of the Bardeen–Cooper–Schrieffer (BCS) theory using parameters derived from ab-initio density functional calculations for electrons and phonons enables us to derive the critical temperature of 1.64 K and an average superconducting gap of 0.25 meV at 0 K. We extend the investigation further into the superconductivity under pressure, showing how *T*_*c*_ and the gap of UTe_2_ change under compression in ways that are consistent with the results of experiment.

## Introduction

Efforts devoted to the research of UTe_2_^1–9^ have revealed very interesting physics about the heavy-fermion superconductor. UTe_2_ has a critical temperature (*T*_*c*_) of 1.6 K and high anisotropic upper critical fields and reentrant superconducting phases. A small superconducting gap^2^ around 0.25 meV has been extracted from scanning tunneling microscopy under zero magnetic field. Under pressure, *T*_*c*_ has been raised to values around 3 K^7^. Most intriguingly, UTe_2_ is generally considered a nearly ferromagnetic spin-triplet superconductor, even though the associated magnetism is intuitively incompatible with superconductivity. In this paper we present a theoretical approach to explain the superconducting mechanism of UTe_2_ in terms of exchange interaction between electrons, trying in the process to broaden the understanding and application of the strong-correlation physics behind.

A strong indication to the direction of investigation comes from the iron-based superconductor, which is also associated with magnetism and has spin fluctuation as its suggested superconducting mechanism. In the well-known family of FeTe_1−*x*_Se_*x*_, electrons with opposite spins form singlet Cooper pairs $\left( {\left| \uparrow \right\rangle \left| \downarrow \right\rangle - \left| \downarrow \right\rangle \left| \uparrow \right\rangle } \right)/\sqrt 2$ and induce an *s*-wave superconducting gap, where $\uparrow$ ($\downarrow$) indicates spin up (down) direction. In the particular example of FeTe_0.55_Se_0.45_^10^, non-trivial topological surface states have been detected experimentally along with the associated topological superconductivity. Theoretical research on the more accessible FeTe_0.5_Se_0.5_ not only produces accurate surface states^11,12^ but provides evidence that exchange interaction^12^ between electrons is driving the superconductivity, under ambient or higher pressures^13^. An extension of the theory to UTe_2_ follows much the same reasoning, as one spin-triplet configuration—$\left( {\left| \uparrow \right\rangle \left| \downarrow \right\rangle + \left| \downarrow \right\rangle \left| \uparrow \right\rangle } \right)/\sqrt 2$—consists also of two electrons having opposite spins, and therefore antisymmetric spatial wavefunction.

## Results and discussion

### UTe_2_ in normal ground state

**Figure 1:**
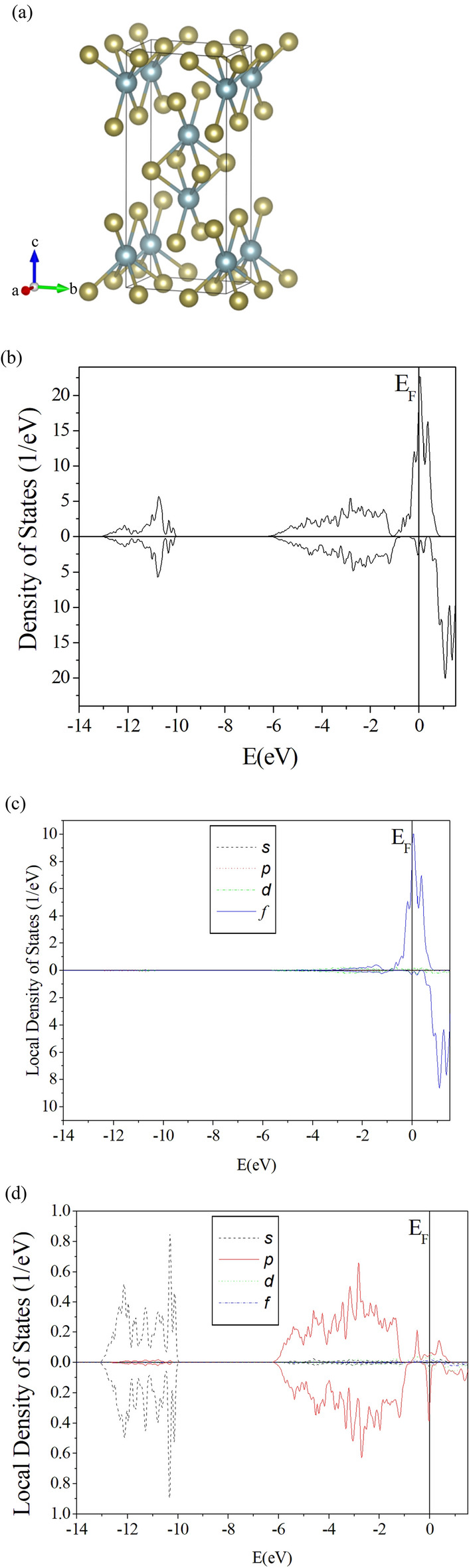
(**a**) UTe_2_ crystal and the three mutually perpendicular axes, with azure (olive) spheres representing U (Te) atoms. (**b**) DOS associated with the majority spin (Top) and minority spin (bottom) of UTe_2_. (**c**) Spin-polarized LDOS in partial waves for a U atom in UTe_2_. Top (bottom) panel is for the majority (minority) spin. (**d**) Spin-polarized LDOS in partial waves for a Te atom in UTe_2_. Top (bottom) panel is for the majority (minority) spin.

UTe_2_ maintains the orthorhombic crystal structure in the space group *Immm* down to the temperature of 2.7 K, according to one recent experiment^14^ using low-temperature neutron diffraction. The structure is shown in Fig. 1a with its experimentally determined lattice constants *a* = 4.123 Å, *b* = 6.086 Å and *c* = 13.812 Å along the three perpendicular axes, which are adopted for our calculation of the electronic structure of UTe_2_.

Total density of states (DOS) from 36 energy bands are presented in the top panel of Fig. 1b for the majority spin and bottom panel for the minority spin. The figure clearly shows that UTe_2_ in normal state is paramagnetic and capable of undergoing ferromagnetic fluctuation^9^, with DOS corresponding to the majority spin far exceeding DOS associated with the minority spin near the Fermi level. Local density of states (LDOS) in partial waves for one U atom are presented in Fig. 1c, attributing the magnetism overwhelmingly to the spin splitting of *f* orbitals near the Fermi level. Sharp peaks of LDOS also point to concentration of nearly flat energy bands contributed by U atoms around the Fermi level. In contrast, Te atoms have negligible participation in the conduction of UTe_2_, with most electrons distributed in lower energies in either *p* or *s* waves as illustrated in Fig. 1d. Overall picture of the density functional calculation is very consistent with previous theoretical calculations^8,15^. There is no doubt that interaction between electrons contributed by U ions is the major source of superconductivity.

### BCS model for UTe_2_ in the superconducting state

The BCS model^16,17^ is formulated by a Hamiltonian *H* consisting of two terms associated with one-particle interaction and two-particle interaction separately,

1 $$ H = \sum\limits_{{{\mathbf{k}},m_{s} }} {\varepsilon_{{\mathbf{k}}} } a_{{{\mathbf{k}},m_{s} }}^{ + } a_{{{\mathbf{k}},m_{s} }} - \sum\limits_{{{\mathbf{k}},{\mathbf{k^{\prime}}}}} {V_{{{\mathbf{kk^{\prime}}}}} a_{{{\mathbf{k}} \uparrow }}^{ + } } a_{{ - {\mathbf{k}} \downarrow }}^{ + } a_{{ - {\mathbf{k^{\prime}}} \downarrow }} a_{{{\mathbf{k^{\prime}}} \uparrow }} , $$

where $a_{{{\mathbf{k}},m_{s} }}^{ + }$ ($a_{{{\mathbf{k}},m_{s} }}$) is the creation (annihilation) operator for the quasi-particle with crystal momentum **k** and spin *m*_*s*_ ($\uparrow$ for up and $\downarrow$ for down) and $\varepsilon_{{\mathbf{k}}}$ in the first term is the associated one-particle energy derived from the mean field. The second term describes the attractive two-particle interaction between electrons in the Cooper pairs. Formation of triplet Cooper pairs is driven by the exchange interaction $- V_{{{\mathbf{kk^{\prime}}}}}$ as expressed in the following equation,

2 $$ E_{t} - E_{s} = - 2\iint {\psi_{{\mathbf{k}}}^{ * } }({\mathbf{r}})\psi_{{{\mathbf{k^{\prime}}}}}^{ * } ({\mathbf{r^{\prime}}})\frac{{e^{2} }}{{{\upvarepsilon }\left| {{\mathbf{r}} - {\mathbf{r^{\prime}}}} \right|}}\psi_{{\mathbf{k}}} ({\mathbf{r^{\prime}}})\psi_{{{\mathbf{k^{\prime}}}}} ({\mathbf{r}})d^{3} rd^{3} r^{\prime} = - 2V_{{{\mathbf{kk^{\prime}}}}} , $$

where *E*_t_ (*E*_s_) is the total energy of the two-electron system at triplet (singlet) state and $\psi_{{\mathbf{k}}} ({\mathbf{r}})$ is the Bloch wave function corresponding to an electron with crystal momentum **k**. The two-particle interaction $e^{2} /{\upvarepsilon }\left| {{\mathbf{r}} - {\mathbf{r^{\prime}}}} \right|$, with *e* the absolute value of the electron charge and ${\upvarepsilon }$ the dielectric constant, indicates its origin in screened Coulomb interaction. The screening is caused mainly by interacting electrons, influenced quite limitedly by collective motions of ions (phonons). The momentum-dependent gap parameter $\Delta_{{\mathbf{p}}}$ at 0 K is defined for the ground state $\left| G \right\rangle$ by $\Delta_{{\mathbf{p}}} = \sum\limits_{{\mathbf{k}}} {V_{{{\mathbf{kp}}}} } \left\langle G \right|a_{{{\mathbf{k}} \uparrow }}^{ + } a_{{ - {\mathbf{k}} \downarrow }}^{ + } \left| G \right\rangle$, which, after further derivation, can be expressed as

3 $$ \Delta_{{\mathbf{p}}} = \sum\limits_{{\mathbf{k}}} {V_{{{\mathbf{kp}}}} \frac{{\Delta_{{\mathbf{k}}} }}{{2E({\mathbf{k}})}}} , $$

where $E({\mathbf{k}}) = \sqrt {(\varepsilon_{{\mathbf{k}}} - \mu )^{2} + \Delta_{{\mathbf{k}}}^{2} }$ is the quasi-particle energy adjusted for chemical potential μ.

In practice an average exchange energy $- V$ is extracted from first-principles density functional calculations of UTe_2_ as described in “Methods” section by considering only electrons separated by nearest neighbors of U, which dominate the contributions to exchange interaction. The value of *V* turns out to be 18.0 meV. Strong screening of electrons greatly diminishes terms between second-nearest neighbors and those beyond. As a result of this approximation $V_{{{\mathbf{kp}}}}$ is replaced by *V* and can be taken out of the summation over **k** in the first Brillouin zone, leading to further simplification by using an average superconducting gap $\Delta$ in (3). The adoption of a constant $\Delta$ is bolstered by a latest experiment claiming that UTe_2_ is a fully gapped superconductor without line or point nodes^18^. Even in a multi-gap scenario, as some experiments claim^3,19^, the summation over** k** points in (3) tends to even out the differences between the gaps and the use of an average $\Delta$ may still be a good approximation.

**Figure 2:**
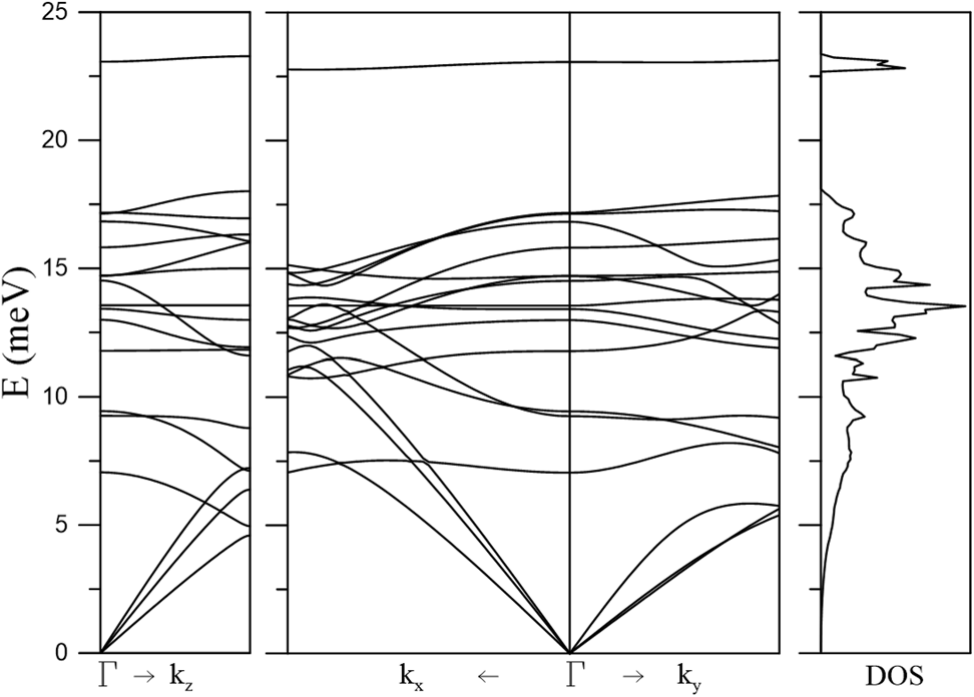
Phonon dispersion curves along three axes and associated DOS of UTe_2_.

Derivation of *T*_*c*_ and $\Delta$ follows by transforming the summation over **k** in (3) into an integration over energy values between $\mu - \hbar \omega_{D}$ and $\mu + \hbar \omega_{D}$, taking into account the phonon contribution represented by the Debye frequency $\omega_{D}$ of UTe_2_. Shown in Fig. 2 are phonon dispersion curves of UTe_2_ along the *a*, *b*, and *c* directions and the associated total density of phonon states, including three acoustic modes and 15 optical modes for each **k** point in the first Brillouin zone. The value of $\hbar \omega_{D}$ is found to be 17.8 meV from the Debye model and the calculated phonon dispersion relation.

*T*_*c*_ is derived from $1.13\Theta_{D} e^{ - 1/\lambda }$, where Debye temperature $\Theta_{D}$ is $\hbar \omega_{D}$ divided by the Boltzmann’s constant and *λ* is the product of *V* and the density of states $N(\varepsilon_{F} )$ for a single spin at the Fermi level. $\Theta_{D}$ and $N(\varepsilon_{F} )$ are calculated to be 206.6 K and 11.2/eV respectively, giving a *T*_*c*_ of 1.64 K very close to the experimental value. The relatively small value of *λ*, equal to 0.20, indicates a weak-coupling nature and suitability of the formula for *T*_*c*_. The superconducting gap corresponding to the ground state $\Delta (0) = 2\hbar \omega_{D} e^{ - 1/\lambda }$ is 0.250 meV. The calculated $\Delta (0)$ is also very close to the experiment employing scanning tunneling microscopy^2^ as previously mentioned, which shows the same size of gap at the lowest temperature.

### UTe_2_ under pressure

**Figure 3:**
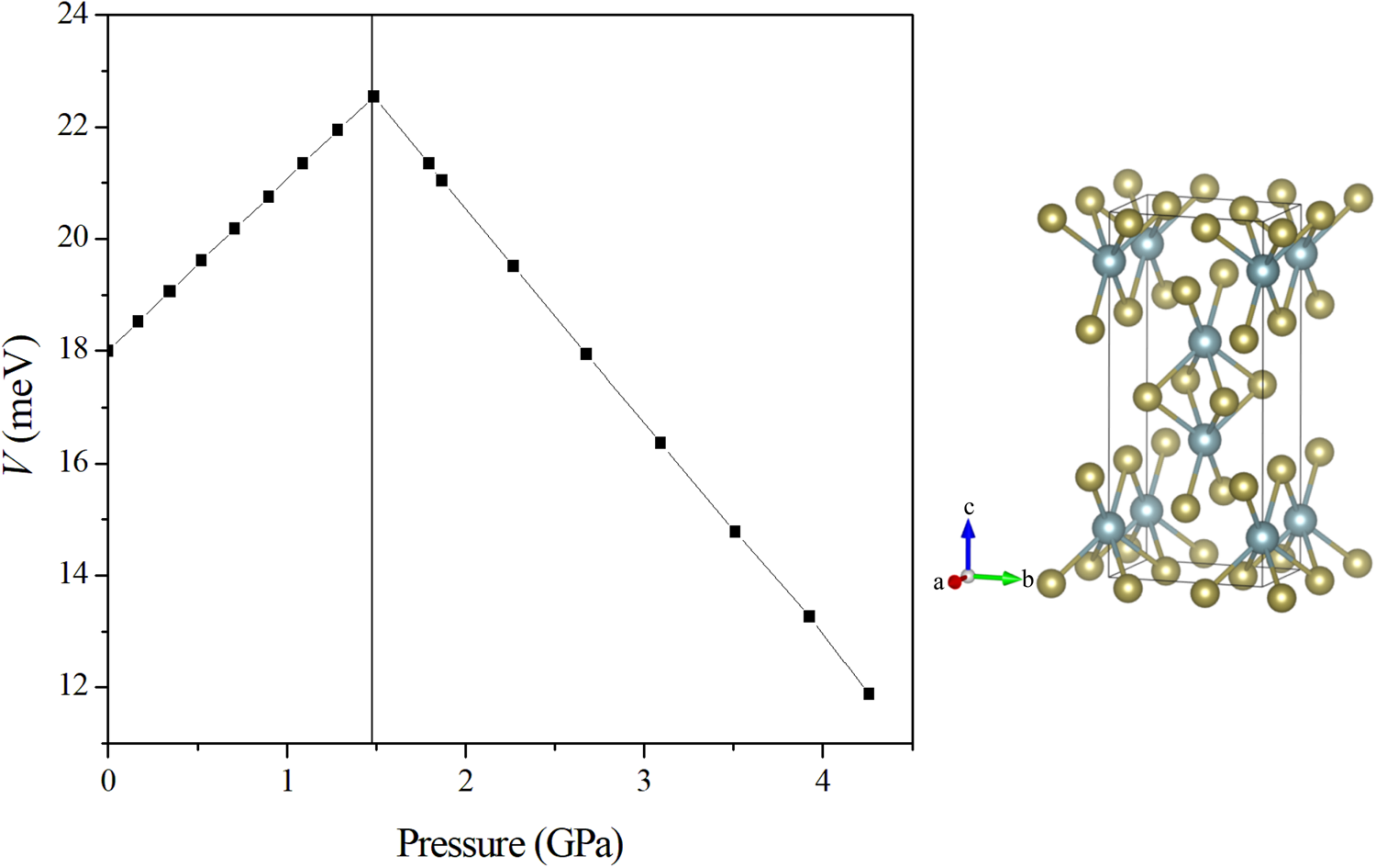
Variation of *V* as UTe_2_ is compressed solely in the *c* direction (left panel) to 1.485 GPa and then uniformly pressured to 4.257 GPa along *a* and *b* (right panel).

Changes of *T*_*c*_ and $\Delta (0)$ depend heavily on the direction of compressive force exerted on UTe_2_. Following the revelation that the most significant structural changes over temperature variation are U ion positions and U–U distance along the *c* direction^14^, our investigation starts with the uniaxial compression along the *c* axis. The left part of Fig. 3 plots the rising exchange interaction *V* with respect to the increase of pressure solely in the *c* direction, reaching 22.5 meV and a strain of − 2% at 1.485 GPa. The rising curve reinforces the argument that nearest neighbors of U along *c* direction dominates the contribution of exchange interaction. Pressure exerted along *a* and *b* directions, however, leads to the decrease of *V*, as depicted in the right part of Fig. 3 continuing from 1.485 to 4.257 GPa by uniform strains along both *a* and *b* directions. An apparent cause of the decline of *V* is the increase of Coulomb repulsion and strengthening of screening effect, which greatly suppresses the exchange interaction between electrons.

**Figure 4:**
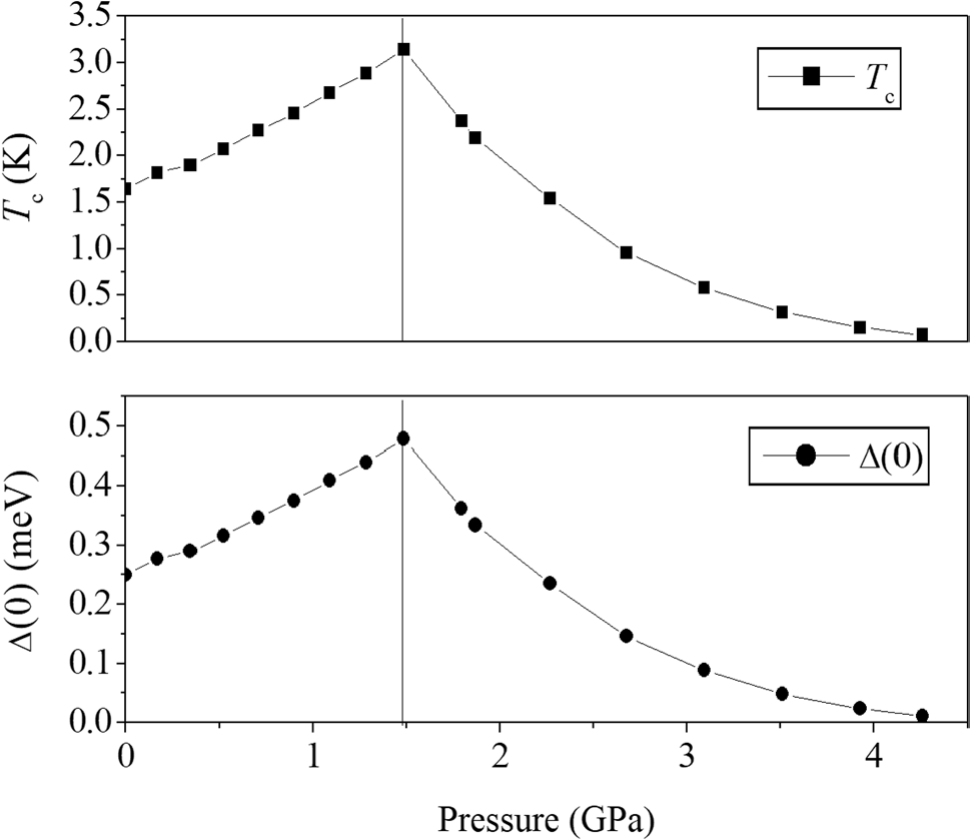
Variation of *T*_*c*_ and Δ(0) as UTe_2_ is compressed solely in the *c* direction (left panels) to 1.485 GPa and then uniformly pressured to 4.257 GPa along *a* and *b* (right panels).

Variations of *T*_*c*_ and $\Delta (0)$ over the same range of pressure as shown in Fig. 3 are separately plotted in Fig. 4. *T*_*c*_ and $\Delta (0)$ increase under uniaxial compression along the *c* direction, reaching 3.14 K and 0.478 meV at 1.485 GPa, mainly because of the rapid rise of *V* and much milder but continuing decrease of $N(\varepsilon_{F} )$ (− 8.52% at 1.485 GPa). $\Theta_{D}$ persistently goes higher due to the stiffened elastic constant under pressure, but its accumulated increase is still less than 2% at the end of uniaxial compression. Beginning with uniform compression along *a* and *b* axes, the right parts of Fig. 4 describe an exponential decline of both *T*_*c*_ and $\Delta (0)$ as a result of the sharp downturn of *V* and relatively steady value of $N(\varepsilon_{F} )$. Superconductivity essentially disappears above 4 GPa.

The two-panel presentations regarding pressure directions in both Figs. 3 and 4 provide a practical guide for analysis of an actual compression in experiment, which can be exerted along any direction and hence has a combination of effects from both. The left panels in the two figures starts with ambient pressure and ends at 1.485 GPa, indicating a path of pushing *T*_*c*_ up to 3.14 K, which is a reasonable increase comparable to that reported in the experiment^7^. The right panels indicate that further compression along the *a* and *b* directions can only lead to the decline of *V*, *T*_*c*_, and $\Delta (0)$. It is conceivable that in an experiment a single crystal of UTe_2_ is subject to compression from different directions simultaneously. There is therefore a possible competition between an increase of *V* from compression along *c* and decrease of *V* along *a* and *b*, contributing to possible phases of superconductivity. Our calculation is also consistent with a latest experiment^20^ showing a particular sensitivity of superconductivity of UTe_2_ to deformations along the *c* axis. The anisotropic response of *T*_*c*_ to compression may also be viewed from the perspective of quasi-one-dimensional bands in multi-band systems^21–23^, which may have a similar connection to the critical magnetic field as well.

In summary, we have studied the superconductivity of UTe_2_ by using the BCS model adapted to accommodate spin-triplet Cooper pairs, with the exchange interaction as the driving force and key parameters derived from first-principles density functional calculations. The method produces accurate *T*_*c*_ and $\Delta (0)$ for the ground state. It also maps out their variation with respect to compression of different magnitudes and directions. The study, along with previous ones for singlet superconductivity, suggests that more superconductors can be explored through the consideration of exchange interaction.

## Methods

The normal ground state and phonon bands of UTe_2_ were derived from first-principles density functional calculations based on the VASP code^24,25^. Projector augmented-wave pseudopotentials and the general gradient approximation (GGA)^26^ were employed with an energy cutoff at 500 eV. The primitive cell of UTe_2_ contains two U and four Te atoms and a 7 × 7 × 7 Monkhorst–Pack **k**-point sampling was adopted.

Dynamics of the creation and annihilation operators can be obtained from the two relations $i\hbar \frac{d}{dt}a_{{{\mathbf{p}} \uparrow }}^{ + } = \left[ {a_{{{\mathbf{p}} \uparrow }}^{ + } ,H} \right]$ and $i\hbar \frac{d}{dt}a_{{ - {\mathbf{p}} \downarrow }}^{{}} = \left[ {a_{{ - {\mathbf{p}} \downarrow }}^{{}} ,H} \right]$, where *H* is the full BCS Hamiltonian described in the main text. By calculating the two commutation relations we obtain a pair of coupled equations of motion for the operators,

4 $$ i\hbar \frac{d}{dt}a_{{{\mathbf{p}} \uparrow }}^{ + } = - \varepsilon_{{\mathbf{p}}} a_{{{\mathbf{p}} \uparrow }}^{ + } + \Delta_{{\mathbf{p}}} a_{{ - {\mathbf{p}} \downarrow }}^{{}} $$

and

5 $$ i\hbar \frac{d}{dt}a_{{ - {\mathbf{p}} \downarrow }}^{{}} = \varepsilon_{{\mathbf{p}}} a_{{ - {\mathbf{p}} \downarrow }}^{{}} + \Delta_{{\mathbf{p}}} a_{{{\mathbf{p}} \uparrow }}^{ + } , $$

with $\Delta_{{\mathbf{p}}} = \sum\limits_{{\mathbf{k}}} {V_{{{\mathbf{kp}}}} } \left\langle G \right|a_{{{\mathbf{k}} \uparrow }}^{ + } a_{{ - {\mathbf{k}} \downarrow }}^{ + } \left| G \right\rangle$ and $\left| G \right\rangle$ representing the superconducting ground state.

Taking the time dependence of the operators as proportional to $e^{ - i\varepsilon t/\hbar }$ (4) and (5) are converted into an eigenvalue problem

6 $$ \left( {\begin{array}{*{20}c} { - \varepsilon_{{\mathbf{p}}} } & {\Delta_{{\mathbf{p}}} } \\ {\Delta_{{\mathbf{p}}} } & {\varepsilon_{{\mathbf{p}}} } \\ \end{array} } \right)\left( {\begin{array}{*{20}c} {a_{{{\mathbf{p}} \uparrow }}^{ + } } \\ {a_{{ - {\mathbf{p}} \downarrow }}^{{}} } \\ \end{array} } \right) = \varepsilon \left( {\begin{array}{*{20}c} {a_{{{\mathbf{p}} \uparrow }}^{ + } } \\ {a_{{ - {\mathbf{p}} \downarrow }}^{{}} } \\ \end{array} } \right), $$

which returns the quasiparticle energy $E_{{\mathbf{p}}} = \varepsilon = \sqrt {\varepsilon_{{\mathbf{p}}}^{2} + \Delta_{{\mathbf{p}}}^{2} }$ and two quasiparticle operators $\gamma_{{{\mathbf{p}} \uparrow }}^{ + }$ and $\gamma_{{ - {\mathbf{p}} \downarrow }}$ as linear combinations of $a_{{{\mathbf{p}} \uparrow }}^{ + }$ and $a_{{ - {\mathbf{p}} \downarrow }}^{{}}$

7 $$ \gamma_{{{\mathbf{p}} \uparrow }}^{ + } = u_{{\mathbf{p}}} a_{{{\mathbf{p}} \uparrow }}^{ + } - v_{{\mathbf{p}}} a_{{ - {\mathbf{p}} \downarrow }}^{{}} ; $$

8 $$ \gamma_{{ - {\mathbf{p}} \downarrow }} = v_{{\mathbf{p}}} a_{{{\mathbf{p}} \uparrow }}^{ + } + u_{{\mathbf{p}}} a_{{ - {\mathbf{p}} \downarrow }}^{{}} . $$

The values of $u_{{\mathbf{p}}}$ and $v_{{\mathbf{p}}}$ are respectively $\sqrt {(1 + \varepsilon_{{\mathbf{p}}} /E_{{\mathbf{p}}} )/2}$ and $\sqrt {(1 - \varepsilon_{{\mathbf{p}}} /E_{{\mathbf{p}}} )/2}$, and are used to construct the superconducting ground state $\left| G \right\rangle = \prod\limits_{{\mathbf{k}}} {(u_{{\mathbf{k}}} } + v_{{\mathbf{k}}} a_{{{\mathbf{k}} \uparrow }}^{ + } a_{{ - {\mathbf{k}} \downarrow }}^{ + } {)}\left| {0} \right\rangle$ from the vacuum $\left| {0} \right\rangle$, where the product includes all **k** points in the first Brillouin zone. It is now ready to calculate $\left\langle G \right|a_{{{\mathbf{k}} \uparrow }}^{ + } a_{{ - {\mathbf{k}} \downarrow }}^{ + } \left| G \right\rangle$ by using the transformation in (7) and (8), obtaining $\left\langle G \right|a_{{{\mathbf{k}} \uparrow }}^{ + } a_{{ - {\mathbf{k}} \downarrow }}^{ + } \left| G \right\rangle = \left\langle G \right|u_{{\mathbf{k}}} v_{{\mathbf{k}}} \gamma_{{ - {\mathbf{k}} \downarrow }} \gamma_{{ - {\mathbf{k}} \downarrow }}^{ + } \left| G \right\rangle = u_{{\mathbf{k}}} v_{{\mathbf{k}}} = \frac{{\Delta_{{\mathbf{k}}} }}{{2E_{{\mathbf{k}}} }}$, so that $\Delta_{{\mathbf{p}}}$ in (4), (5), and (6) can be expressed as

9 $$ \Delta_{{\mathbf{p}}} = \sum\limits_{{\mathbf{k}}} {V_{{{\mathbf{kp}}}} } \frac{{\Delta_{{\mathbf{k}}} }}{{2\sqrt {(E_{{\mathbf{k}}} - \mu )_{{}}^{2} + \Delta_{{\mathbf{k}}}^{2} } }}, $$

where the summation is also over the Brillouin zone and quasiparticle energy $E_{{\mathbf{k}}}$ is adjusted for chemical potential $\mu$.

Adoption of the average *V* and Δ for $V_{{{\mathbf{kp}}}}$ and $\Delta_{{\mathbf{k}}}$, for reasons explained in the main text, greatly reduces the derivation of Δ (at 0 K) by transforming (9) into an integration over the energy levels between $\mu - \hbar \omega_{D}$ and $\mu + \hbar \omega_{D}$

$$ 1/V = \int_{{\mu - \hbar \omega_{D} }}^{{\mu + \hbar \omega_{D} }} {N(\varepsilon )\frac{1}{{2\sqrt {(\varepsilon - \mu )^{2} + \Delta^{2} } }}} d\varepsilon = \int_{{ - \hbar \omega_{D} }}^{{\hbar \omega_{D} }} {N(\varepsilon + \mu )\frac{1}{{2\sqrt {\varepsilon^{2} + \Delta^{2} } }}} d\varepsilon , $$

where *N*(**ε**) is the DOS for a single spin. As $\hbar \omega_{D}$ is tiny compared with the spread of electronic energy levels, $N(\varepsilon + \mu )$ is replaced by $N(\varepsilon_{F} )$ in approximation and taken out of the integration, giving the result $\Delta (0) = \frac{{\hbar \omega_{D} }}{\sinh (1/\lambda )}$ with $\lambda = VN(\varepsilon_{F} )$. For weak coupling (small value of λ) the expression is further simplified to $\Delta (0) = 2\hbar \omega_{D} e^{ - 1/\lambda }$.

At finite temperature *T* thermal average must be taken for the calculation:

10 $$ \begin{aligned} & \left\langle {a_{{{\mathbf{k}} \uparrow }}^{ + } a_{{ - {\mathbf{k}} \downarrow }}^{ + } } \right\rangle = u_{{\mathbf{k}}} v_{{\mathbf{k}}} \left\langle {1 - \gamma_{{{\mathbf{k}} \uparrow }}^{ + } \gamma_{{{\mathbf{k}} \uparrow }} - \gamma_{{ - {\mathbf{k}} \downarrow }}^{ + } \gamma_{{ - {\mathbf{k}} \downarrow }} } \right\rangle = u_{{\mathbf{k}}} v_{{\mathbf{k}}} (1 - \frac{2}{{e^{{E_{{\mathbf{k}}} /k_{B} T}} + 1}}) = \frac{{\Delta_{{\mathbf{k}}} (T)}}{{2E_{{\mathbf{k}}} }}\tanh \frac{{E_{{\mathbf{k}}} }}{{2k_{B} T}}, \\ & {\text{leading to}}\;\Delta_{{\mathbf{p}}} (T) = \sum\limits_{{\mathbf{k}}} {V_{{{\mathbf{kp}}}} } \frac{{\Delta_{{\mathbf{k}}} (T)}}{{2E_{{\mathbf{k}}} }}\tanh \frac{{E_{{\mathbf{k}}} }}{{2k_{B} T}}. \\ \end{aligned} $$

Converting (10) into an integration with the same approximations and adjustment of energy as in (9), we obtain $1/V = N(\varepsilon_{F} )\int_{0}^{{\hbar \omega_{D} }} {\frac{1}{{\sqrt {\varepsilon^{2} + \Delta (T)^{2} } }}\tanh \frac{{\sqrt {\varepsilon^{2} + \Delta (T)^{2} } }}{{2k_{B} T}}} d\varepsilon$. Since $\Delta (T_{c} ) = 0$, *T*_*c*_ can be extracted from the integration $1 = \lambda \int_{0}^{{\hbar \omega_{D} }} {\frac{1}{\varepsilon }\tanh \frac{\varepsilon }{{2k_{B} T_{c} }}} d\varepsilon$, resulting in the equation $T_{c} = 1.13\Theta_{D} e^{ - 1/\lambda }$.

## Data Availability

The datasets used and/or analyzed during the current study are available from the corresponding author on reasonable request.
